# A Multiplex PCR System of Novel Microsatellite Loci for Population Genetic Application in Walnuts

**DOI:** 10.3390/plants12244101

**Published:** 2023-12-07

**Authors:** Zuo-Ying Xiahou, Moses C. Wambulwa, Zu-Chang Xu, Lin-Jiang Ye, Peng-Zhen Fan, Ephie A. Magige, Ya-Huang Luo, Jie Liu

**Affiliations:** 1 CAS Key Laboratory for Plant and Biodiversity of East Asia, Kunming Institute of Botany, Chinese Academy of Sciences, Kunming 650201, China; xiahouzuoying@mail.kib.ac.cn (Z.-Y.X.); mcwambulwa@gmail.com (M.C.W.);; 2 Germplasm Bank of Wild Species, Kunming Institute of Botany, Chinese Academy of Sciences, Kunming 650201, China; 3 University of the Chinese Academy of Sciences, Beijing 100049, China; 4 Department of Life Sciences, School of Science and Computing, South Eastern Kenya University, Kitui 170-90200, Kenya; 5 Key Laboratory of Plant Resources and Biodiversity of Jiangxi Province, Jingdezhen University, Jingdezhen 333400, China

**Keywords:** genetic diversity, *Juglans*, microsatellite marker, multiplex PCR, transferability, walnut

## Abstract

Multiplex polymerase chain reaction (PCR) of microsatellite loci allows for simultaneous amplification of two or more pairs of primers in a single PCR reaction; hence, it is cost and time effective. However, very few attempts have been reported in non-model species. In this study, by combining a genome-based de novo development and cross-species application approach, a multiplex PCR system comprising 5 PCR reactions of 33 microsatellites consisting of 26 novel genomic and 7 literature-sourced loci was tested for polymorphisms, cross-species transferability, and the ability to assess genetic diversity and population structure of three walnut species (*Juglans* spp.). We found that the genome-based approach is more efficient than other methods. An allelic ladder was developed for each locus to enhance consistent genotyping among laboratories. The population genetic analysis results showed that all 33 loci were successfully transferred across the three species, showing high polymorphism and a strong genetic structure. Hence, the multiplex PCR system is highly applicable in walnut species. Furthermore, we propose an efficient pipeline to characterize and genotype polymorphic microsatellite loci. The novel toolbox developed here will aid future ecology and evolution studies in walnut and could serve as a model for other plant species.

## 1. Introduction

The genus *Juglans* comprises more than 21 monoecious, wind-pollinated, and deciduous tree species [1]. These species are valued for their nutritious nuts, high-quality timber, and medicinal significance [2,3]. While *J. regia* has a wide distribution range spanning from Europe to East Asia, *J. cathayensis*, *J*. *sigillata*, and *J*. *mandshurica* are mainly distributed in China, although *J. mandshurica* has also been reported in Korea, Japan, and far eastern Russia [4,5].

Morphological, agronomic, biochemical, and molecular (DNA-based) approaches have been applied in walnut cultivar identification, analysis of phylogenetic relationships, genetic diversity studies, and germplasm characterization [6]. While morphological and agronomic characteristics are greatly affected by growth and environmental conditions, hence being prone to inaccuracies, molecular markers are more robust and independent of developmental or environmental factors. Certain DNA-based tools, such as restriction fragment length polymorphism (RFLP), amplified fragment length polymorphism (AFLP), randomly amplified fragment polymorphic DNA (RAPD), and inter-simple sequence repeat (ISSR), may also have limitations of being dominant and labor-intensive. Microsatellites, or short tandem repeats (SSRs), have been described as desirable DNA-based markers due to their abundance in the genome, high polymorphism, and co-dominant inheritance [7]. However, SSRs also have limitations, such as null alleles, which can hinder accurate population genetic analysis.

Traditional methods of SSR development are complex and time- and labor-intensive owing to the series of complicated steps involved in the construction of genomic libraries, primer design, and PCR amplification. To improve the efficiency of SSR development, White and Powell [8] significantly improved the success rate by optimizing the protocol for combining biotin with magnetic beads. Subsequently, Zane et al. [9] developed fast, simple, and efficient FIASCO (fast isolation of sequences containing repeats)-based microsatellites, which further improved amplification efficiency and reduced the cost of microsatellite development. With the emergence and rapid development of sequencing technologies, sequence data have become more available, resulting in the development of SSR markers based on expressed sequence tags (ESTs). EST-SSRs result from transcribed DNA sites and are therefore more conserved, meaning that they are less polymorphic than genomic SSRs [10], which have a wider distribution throughout the genome, hence providing better map coverage. More recently, with the rapid development of next-generation sequencing technologies, numerous high quality SSRs can be developed by comparing the whole-genome sequences of multiple individuals [11].

Despite the huge strides made in the improvement of SSR markers, their utility has largely been limited by the slow pace of data output with the use of single primer pairs per PCR run. Nevertheless, advances in molecular marker technology have given rise to multiplex PCR for DNA amplification. Multiplex PCR entails simultaneous amplification of more than one SSR locus in a single PCR reaction, coupled with the employment of fluorescence-based automated DNA detection and fragment sizing, making the process time- and cost-effective, in addition to allowing for accurate acquisition of data [12]. Additionally, the standardization of multiplex PCR is guaranteed by the uniform reaction conditions (the amount of template and other PCR components), as well as the minimal variations in PCR across loci [13].

Multiplex PCR is a versatile technique with numerous applications, including disease diagnosis [14], species identification [15], and expression profiling [16], among others. The high-throughput capacity of multiplex PCR has led to its expanding utility in plant population genetics research. For instance, Koh et al. [17] developed a multiplex PCR system for the rapid identification of *Brassica* species, which has proven to be useful for managing germplasm collections. In addition, another innovative approach, introduced by Zhang et al. [18], is target SNP-seq, which combines the advantages of multiplex PCR and high-throughput sequencing for SNP genotyping. The method was successfully employed to construct a DNA fingerprint of 261 cucumber varieties. Additionally, Yao et al. [19] utilized multiplex PCR technology to establish a comprehensive molecular marker selection system for wheat, which further facilitated the selection of strong gluten wheats.

In walnut, microsatellite loci have been developed for different species in previous studies (Table S1) [20,21,22,23]. However, multiplexing of microsatellite loci has not yet been fully embraced in *Juglans*, with only a few published studies [24]. Hence, the goal of the present study was to develop and characterize highly polymorphic SSRs by cross-species amplification and de novo development and ultimately to construct a multiplex PCR system for the loci. Furthermore, we validated cross-species transferability and potential for population genetic application in three *Juglans* species (*J. regia*, *J. sigillata*, and *J. cathayensis*). Finally, we propose a pipeline to develop and genotype high polymorphic microsatellite loci for other plants.

## 2. Results

### 2.1. Primer Screening, Development and Selection

From the genomic data of the two *J*. *regia* individuals [25,26], QDD_v3 identified 279,712 SSR loci, of which 39,741 loci were polymorphic in both genomes. We then designed a total of 14,217 primers from these polymorphic SSRs. After careful selection of both newly developed and literature-based primers, we obtained a total of 1055 pairs of primers (568 from the literature and 487 newly developed), each bearing a unique repeat motif (Table S2). Among the 568 primer pairs from the literature, 156 pairs were developed through the FIASCO method, 176 pairs were derived from EST-SSRs, 179 pairs were acquired via the genomic library enrichment method, and 57 pairs were characterized using the NGS (next generation sequencing) method. After three rounds of primer screening, we settled on 118 pairs of primers showing cross-species amplification (Table S3). These pairs comprised 67 primer pairs from literature sources (13 FIASCO, 35 EST-SSR, 17 genomic library enrichment, and 2 NGS) and 51 newly developed primer pairs. Finally, we selected 33 pairs of core primers, which included 7 pairs from the literature (4 EST-based, 2 from genomic library enrichment, and 1 based on NGS), and 26 de novo developed pairs, with none of the FIASCO-based primers selected (Table S4).

### 2.2. Simplex PCR

Simplex PCR yielded primers with an amplification rate in the range of 90–100% for all 95 tested walnut individuals (Table 1). Sixteen primer pairs (12 of which were the newly developed genomic primers) showed a 100% amplification rate. The number of alleles per locus ranged from 4 to 12, with the majority in the range of 5–8. Owing to the small sample sizes of populations XYR (*J. regia*), MGY (*J. cathayensis*), and DTL (*J. mandshurica*), we excluded them from the Hardy–Weinberg equilibrium analysis. A total of 16 primer pairs showed a significant deviation from Hardy-Weinberg equilibrium (HWE) (Table 1).

### 2.3. Multiplex PCR System and Validation

**Figure 1:**
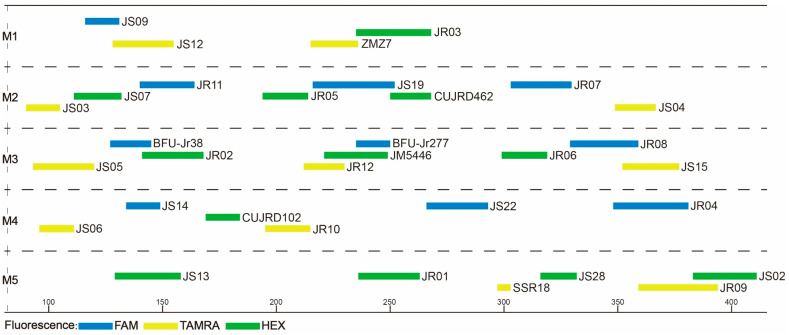
Optimized primer combinations determined by Multiplex Manager. A total of 33 primers were grouped into five multiplex PCR systems (M1–M5). The horizontal scale shows PCR product sizes. The length and color of each bar denote allele range and fluorescent color, respectively.

After a series of readjustments and optimization, the 33 core primer pairs were finally grouped into five categories (M1–M5), each having four, eight, nine, six, and six primer pairs. Of the 33 core primer pairs, a total of 11 primer pairs were redesigned and optimized to suit the m-PCR system (Table S5; Figure 1). Our aim was to combine as many SSR markers as possible in one multiplex PCR. However, due to fluorescent dye and size overlaps, as well as the presence of stutter bands, some polymorphic SSR markers were excluded from multiplexing; hence, 33 core primer pairs were selected as candidates for the multiplex PCR. For convenience, only two fluorescent dyes were used in the M5 group. Due to the disparity in the number of primer pairs per reaction, we used the following proportions: 8 μL 2 × PCR MASTER MIX, 0.2 μL of each primer. With the remaining volume filled up with ddH_2_O, the total reaction volume was 15 μL. The final PCR protocol was as follows: an initial 3-min denaturation at 94 °C, 35 cycles for 30 s at 94 °C, 30 s at different annealing temperatures (Table 2), and 1 min at 72 °C; and a final extension for 5 min at 72 °C. Transferability of these five multiplex systems were gauged based on the amplification rate (Table S6). The amplification rate ranged between 92% and 100% across species (average of 99%), with primer pair JR04 having the lowest amplification rate.

A total of 209 *Juglans* individuals (89 *J. regia*, 60 *J. sigillata,* and 60 *J. cathayensis*) were used to validate the application of the developed m-PCR system. Compared to the simplex PCR, a total of 13 loci, which included 10 newly discovered loci, were amplified with an improved PCR amplification rate (Table S6). In addition, multiplex PCR showed a relatively number of alleles at each locus, although this outcome could be ascribed to the greater number of individuals used in the multiplex system.

### 2.4. Allelic Ladders

Allelic ladders were made for each of the 33 loci to ascertain the accuracy of the amplification results (Figure S1). Systematic errors were identified and rectified by comparing the allelic ladders. Some loci, such as JS19, showed stutter bands, but there was no efficient way to eliminate them; hence, we followed the size information of the loci from the genome to determine the true alleles.

### 2.5. Genetic Diversity and Population Structure Analysis

To test the potential application of the m-PCR system in closely related species, we performed population genetics analysis in three walnut species. The polymorphism information content (*PIC*) ranged from 0.57 (JR10) to 0.85 (JR04), with an average value of 0.72 (Table S6). The number of alleles per locus was between 5 and 15, with primer JR10 showing the lowest value (5) and JS19 the highest (15). Observed heterozygosity (*H*_O_) was lowest in JR01 (0.17) and highest in JS13 (0.68), with an average value of 0.41, while *H*_E_ ranged from 0.38 (JR01) to 1.12 (JR04), with a mean value of 0.83. The average *N*_A_ values for all populations were 3.58, with ranges of 2–5. Observed heterozygosity (*H*_O_) was lowest in population GLR (0.25) and highest in UKR (0.55), while *H*_E_ was lowest in EMC (0.33) and highest in UKR (0.59). The mean values for *H*_O_ and *H*_E_ were 0.41 and 0.47, respectively.

**Figure 2:**
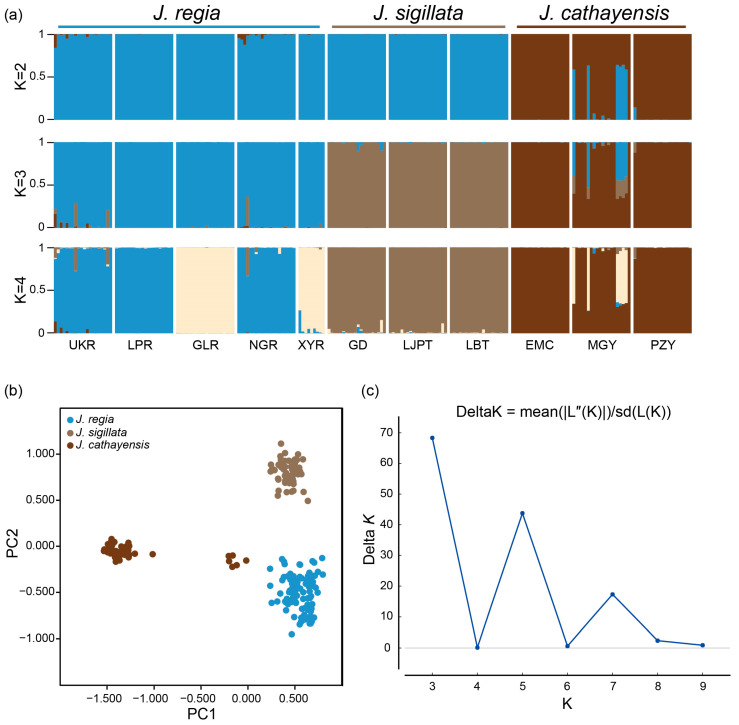
Genetic structure of three walnut species. (**a**) Bayesian clustering of individuals from 11 *Juglans* populations. (**b**) Principal coordinate analysis (PCoA) plot of the three *Juglans* species based on Nei’s genetic distance. Each color represents one morphological species. (**c**) Inference of the optimal *K* value using the Delta*K* (Δ*K*) method.

**Figure 3:**
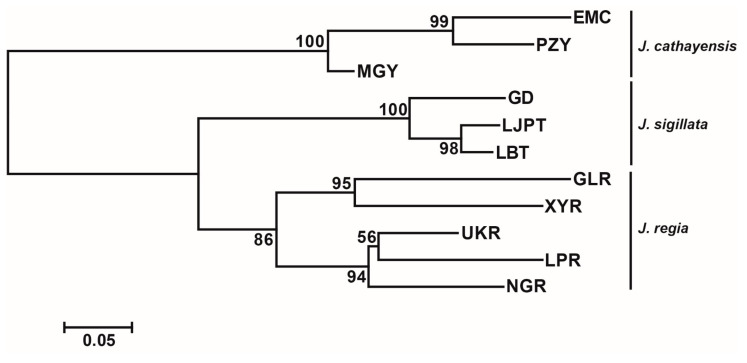
Neighbor joining (NJ) tree based on *F*_ST_ values. The number on the node denotes the bootstrap support value.

Bayesian clustering strongly supported *K* = 3 as the optimal number of clusters (Figure 2a,c). At *K* = 3, there was a clear delineation of the three species, with a clear introgression signal in populations MGY and UKR and minor introgressions in NGR and GD. The first two axes of the PCoA accounted for 27.22% and 12.35% of the genetic variation, respectively. These two axes divided the populations into three groups: *J. regia*, *J. sigillata*, and *J. cathayensis* (Figure 2b). Consistently, the neighbor-joining (NJ) tree grouped the populations into three clusters (Figure 3), further supporting the patterns shown by STRUCTURE and PCoA.

## 3. Discussion

In this study, a multiplex PCR system with an allelic ladder of walnut microsatellite loci was developed, which was successfully validated for population genetic analysis in three walnut species.

### 3.1. Genomic SSR Markers and Transferability

We developed a multiplex SSR system of 33 SSR loci, 26 of which were newly developed based on genomic data. A total of 13 primer pairs showed a 100% amplification rate in simplex PCR reactions (Table S6), indicating the high quality of the SSR markers. Additionally, the m-PCR assay validation showed that 10 genomic primers showed improved amplification rates and could successfully genotype more than 100 walnut individuals. We also observed high levels of polymorphism for the 26 genomic SSR markers in terms of *PIC* and *N*_A_ values, which ranged from 0.57 (JR10) to 0.85 (JR04) for *PIC* and from 5 (JR10) to 15 (JS19) for *N*_A_. These values were higher than those obtained by Vischi et al. [27], although the genomic primers in their study were considered more polymorphic and discriminative compared to EST-SSR markers. It is noteworthy that the selected 33 polymorphic primer pairs comprise a small proportion of primers sourced from the literature (7 of 568). Surprisingly, no FIASCO-based SSR markers were retained after primer selection, indicating that FIASCO may not be a preferred method for SSR marker development in the genomic era, further emphasizing caution when we want to employ published primers using traditional approaches. Our genome-based approach was more successful (26 of 33) relative to other methods (7 of 33) [28,29,30,31]. It is not surprising that our genomic-based SSR markers had a relatively higher amplification rate and polymorphism. Basically, genomic SSRs are derived from introns or intergenic sequences, which tend to have greater variability than exons [32]. Hence, if the primer region is conservative enough, the locus will have a high PCR amplification rate with high polymorphism.

SSR development is time- and labor-intensive; thus, it is important to investigate the transferability of SSR markers among species [33]. The success of marker transferability is dependent upon the evolutionary relationships among the species or genera involved [34], and it is bound to decrease as the evolutionary distance between the source and the target species increases. In the present study, all the SSR markers displayed a high amplification rate across all species, indicating excellent transferability of the markers and suggesting that the selected SSR loci are conserved across the four species. Indeed, SSR marker transferability among walnut species and related genera has also been previously reported [23], suggesting that microsatellites might be conserved across genera within Juglandaceae. Previous studies have demonstrated the intra-generic transferability of SSRs among closely related plant species [33,35], although transferability among distantly related species has also been observed [36].

### 3.2. Polymorphism of the SSR Markers and Genetic Diversity

The selected 33 core primers generally showed a high level of polymorphism (*PIC* = 0.72, *N*_A_ = 9.88, *H*_E_ = 0.83). *PIC* values signify the discriminatory ability of any locus since they represent the number of alleles per locus and their frequencies in a population (basically, a measure of genetic diversity values) [37]. Using a *PIC* threshold between 0.5 and 0.7, we classified 14 loci as informative (*PIC* > 0.5) and 19 loci as suitable for mapping (*PIC* > 0.7). The averages of the *N*_A_ and *PIC* values in our study were higher than those reported earlier for SSR primers in other walnut species [38,39]. This finding might be attributed to the larger number of samples used for genetic diversity analysis in the present study; we used 209 walnut cultivars, while Ikhsan et al. [39] and Zhang et al. [38] used eight and 98 cultivars respectively. The *PIC* values in our study were comparable to those previously reported for *J*. *regia* and *J*. *sigillata* [40]. The average *H*_E_ value was 0.83, which was higher than the values reported by Shavvon et al. [41]. The high polymorphism observed for these markers indicates their suitability for walnut genetic diversity research.

Our STRUCTURE, PCoA, and NJ results showed a strong genetic structure, clearly delineating the three species. These results are consistent with the traditional taxonomic circumscription [42] and recent population genomic findings [43,44]. In addition, N introgression signal was detected between species. Naturally, *J*. *regia* and *J*. *sigillata* readily hybridize, especially when they occur sympatrically [45], making it difficult to distinguish them [46]. On the other hand, interspecific hybridization among *J*. *regia J. mandshurica,* and *J*. *cathayensis* has been demonstrated [47]. Hence, our newly reported m-PCR system is applicable for population genetic analysis. Notably, the m-PCR system developed here was successfully used in several recent studies of walnut genetic diversity [41,48].

### 3.3. Multiplex PCR System

**Figure 4:**
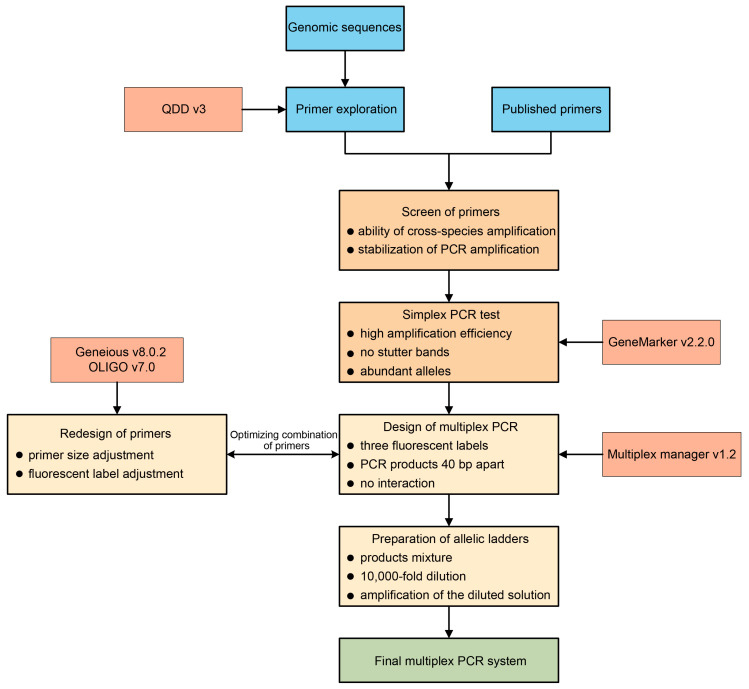
Proposed pipeline for the development of a new m-PCR system. The required software is shown in the boxes with thinner outlines. Each step contains the required screening or inclusion criteria.

Multiplex PCR has been widely employed in various fields, such as disease diagnosis [14], species identification [17], and forensic identification [49]. We aimed to develop a reliable protocol for designing a multiplex PCR system for walnut (Figure 4), with potential application in non-model plant species to ensure standardized results across different laboratories. The use of multiplex PCR can significantly cut down on the resources and time required for analyses; for instance, we successfully compacted 33 PCR reactions into only 5, thereby increasing efficiency several-fold compared to simplex PCR. Although the number of loci that can be included in a multiplex PCR is not strictly limited, some studies have reported up to 26 loci [50]. In our study, we adopted a conservative approach and kept a large distance between primers, resulting in five separate reactions, thus allowing for further optimization of the designed multiplex PCR reactions. The success of PCR amplification directly affected the outcome of our experiments. Indeed, the amplification rate was variable after combining the primers into multiplex PCR products, which could be attributed to the use of different and additional individuals in the multiplex PCR.

Accurate genotyping is crucial for many applications, and the use of an allelic ladder as a reference is critical for achieving this goal [51]. With an allelic ladder, it is possible to quickly identify new genotypes and correct any experimental errors. More importantly, the allelic ladder acts as a common scale across laboratories to genotype the same loci, in turn holding promise for large-scale data sharing and analysis across studies.

## 4. Materials and Methods

### 4.1. Sample Collection and DNA Extraction

Fresh leaves of 212 individuals were collected from 12 populations of 4 walnut species (*J. regia*, *J. sigillata*, *J. cathayensis*, and *J. mandshurica*) distributed across China, Iran, and Pakistan (Table S2) and preserved in silica gel. An individual of *J. sigillata* was used as a control for the PCR amplification. DNA was extracted following a modified cetyltrimethylammonium bromide (CTAB) protocol [52]. The quality and concentration of the DNA were assessed on 1% agarose gel and a NanoDrop^®^ ND-2000 spectrophotometer (Thermo Fisher Scientific, Wilmington, DE, USA), and it was diluted to a concentration of ca. 50 ng/μL for subsequent PCR amplification.

### 4.2. Data Mining and Primer Design

To allow for quality comparisons, we incorporated primers designed by four methods: FIASCO (fast isolation by AFLP of sequences containing repeats), EST (expressed sequence tag), genomic library enrichment, and NGS. FIASCO, EST-based, genomic library enrichment, and NGS by transcriptome data primers were obtained from literature sources (Table S1), while the genomic SSRs (gSSRs) were newly designed in the present study based on two *J. regia* genomes [25,26]. To design new gSSR primers, genomic data of the two *J*. *regia* individuals were downloaded from the National Center for Biotechnology Information (NCBI, https://www.ncbi.nlm.nih.gov/, accessed on 29 July 2016 and 20 December 2017 respectively) database. These genomes were then aligned, followed by the removal of redundant sequences using an identity parameter of 95%. Subsequently, the resultant SSR loci were detected using the bioinformatics program QDD, version 3 [53], and then selected based on their level of polymorphism (at least two alleles). Thereafter, primers were designed from the SSR loci using the QDD program with default parameters. Finally, primers with motif lengths of 3–6 bp, repeat frequencies >6, and PCR product sizes of 100–400 bp were selected for downstream processes.

### 4.3. Primer Screening

To ascertain the quality of the PCR primers, we subjected the primers to three rounds of screening using the protocol described by Xu et al., using the same PCR protocol [23]. Each PCR comprised 18 μL of Golden Star T6 Super PCR Mix (Tsingke Biological Technology, Beijing, China), 0.5 μL of each primer, and 1 μL of DNA template. The following thermocycling regimen was employed: initial denaturation at 95 °C for 5 min, 30 cycles of 95 °C for 10 s, primer annealing temperatures for 3 min, 72 °C for 1 min, and then a final extension at 72 °C for 5 min. The obtained PCR products were analyzed on a 6% polyacrylamide gel (PAGE) to determine the amplification rate. The first step entailed checking whether the loci could be successfully amplified using four individuals of *J*. *sigillata*. The primary objective of the screening was to assess the quality of the loci: repeatability, specificity, and polymorphism of the primers. Repeatability refers to the ability to obtain consistent and reproducible results upon replication, ensuring the reliability of the experimental outcomes. Specificity, on the other hand, pertains to the primer’s capacity to selectively bind to specific positions within the target sequence, ensuring accurate and precise targeting. Additionally, the polymorphisms of the primers were evaluated, whereby a primer pair was deemed polymorphic if it could amplify at least two distinct alleles. Here, we chose 20 individuals of *J*. *sigillata* and four individuals of *J*. *regia*.

To confirm the transferability of the primers across *Juglans* species, we labelled the 5′ end of forward primers with fluorescent dyes (FAM, HEX, or TAMRA; Optimus Bio, Kunming, China) and used them on 95 individuals (60 individuals from 3 populations of *J. sigillata*, 29 individuals of *J. regia*, 3 individuals of *J. cathayenesis*, and 3 individuals of *J. mandshurica*). The size of amplification products and the fluorescent dye were detected by ABI 3730xl DNA analyzer (Applied Biosystems, Thermo Fisher Scientific, Waltham, MA, USA). After the third round of amplification, the primers were filtered based on band clarity, amplification rate, transferability, and allelic polymorphism (at least two alleles). The finally selected primers were classified as ‘core primers’ and preserved for a construct multiplex PCR (m-PCR) system and population genetic testing.

### 4.4. Simplex PCR Amplification

To ensure the quality of amplification after primer screening, we performed simplex PCR amplification using Golden Star T6 Super PCR mix (Beijing Tsingke Biotech Co., Ltd., Beijing, China) since it allows for efficient amplification of fluorescently labeled primers. A total of 95 individuals from seven populations of four species, namely *J. regia*, *J*. *sigillata*, *J. cathayensis*, and *J. mandshurica,* were selected for amplification, with PCR performed in a 20-μL reaction mixture containing 9 μL of 2× PCR MASTER MIX (Beijing Tsingke Biotech Co., Ltd., Beijing, China), 9 μL of ddH_2_O, 0.5 μL of each primer, and 1 μL of DNA template. The PCR protocol was as follows: initial denaturation at 94 °C for 3 min; 30 cycles of 30 s at 94 °C, primer annealing temperature for 30 s, 1 min at 72 °C; and then a final extension at 72 °C for 5 min. We used three fluorescent dyes of unique colors (FAM = blue, HEX = green, TAMRA = yellow). The dye colors and PCR product sizes were distinguished using an ABI 3730xl DNA analyzer, with GeneScan^TM^ 500 LIZ (Applied Biosystems, Thermo Fisher Scientific, Waltham, MA, USA) as an internal size standard. We then determined the amplification rate for each primer pair as described earlier.

### 4.5. Development of m-PCR System and Validation

To improve the efficiency of PCR amplification, we developed an m-PCR amplification system using the selected primers. The Multiplex Manager tool, version 1.2 (http://www.multiplexmanager.com/about.php, accessed on 28 March 2019), was used to design and optimize the m-PCR amplification system, with the selection criteria set as follows: 10 as the maximum number of loci per reaction, 100 as the batch of iteration runs, 7 as the complementarity threshold, and 40 as the minimum distance between loci of the same dye color. To optimize the combinations of the primers, we used Geneious software, version 8.0.2, to redesign the primer sizes and their fluorescent dyes until a stable multiplex PCR primer combination was achieved [54]. We used Oligo software, version 7.0 (DBA Oligo, Inc., Colorado Springs, CO, USA), to redesign the primers [55]. This process was repeated until a satisfactory m-PCR amplification system was attained. We then optimized reaction conditions by conducting gradient experiments on the total volume of reactions (15 μL, 20 μL, and 25 μL), the volumes of primers (0.1 μL, 0.2 μL, 0.3 μL, and 0.5 μL), and the optimal annealing temperature of each m-PCR reaction (set at 1 °C per gradient, with a total of 5 gradients).

To verify the usability of the m-PCR, we sampled 209 individuals from three *Juglans* species (*J. regia*, *J. sigillata,* and *J. cathayensis*), of which 92 individuals were used in the simplex screening step, with another 117 individuals also being used for further population genetic verification. PCR products were genotyped using an ABI 3730xl DNA analyzer.

### 4.6. Development of Allelic Ladders

To improve the genotyping accuracy, we developed an allelic ladder [51] for each of the developed m-PCR primers. First, we selected a few individuals containing all the alleles from the pool of 95 walnut individuals used in the simplex PCR reaction stage. Subsequently, only a few individuals having all the alleles were selected to reduce the failure rate in subsequent amplifications. Then, these individual amplification products were mixed and diluted with double-deionized (dd) water at a ratio of 1:10,000, while ensuring the consistency of each allele. The diluted mixture was then amplified following a simplex PCR procedure, yielding allelic ladders, which were then detected on an ABI 3730xl analyzer.

### 4.7. Data Analysis

Genemarker software, version 2.2.0 (SoftGenetics LLC^®^, State College, PA, USA), was used to visualize and record the amplified PCR product sizes, while CERVUS, version 3.0.7, was used to determine polymorphic information content (*PIC*) values for all the loci [56]. GenAIEx, version 6.5 [57], was used to determine the genetic diversity parameters (number of alleles, *N*_A_; observed heterozygosity, *H*_O_; expected heterozygosity, *H*_E_) at locus and population levels. *PIC* and *N_A_* were used to gain further insights into the level of polymorphism of the selected primers. To infer the genetic structure of the populations, an unrooted tree was generated using the neighbor-joining (NJ) algorithm with 1000 bootstrap replicates in POPTREE, version 2.0 [58]. Briefly, POPTREE builds the NJ tree based on internally computed *F_ST_* (fixation index) estimates. GENEPOP [59] was used to determine departure from Hardy-Weinberg equilibrium. Subsequently, tree topologies were viewed and adjusted in Figtree, version 1.4.4 (http://tree.bio.ed.ac.uk/software/figtree/, accessed on 25 November 2018). Bayesian analysis of population genetic structure with STRUCTURE, version 2.3.4, was used with the admixture model and a correlated allele frequency procedure. To determine the optimal number of clusters (*K*), the population structure was tested at *K* values ranging from 1 to 10, each with 10 replicates based on 100,000 Markov chain–Monte Carlo (MCMC) iterations following a burn-in period of 10,000 steps. Evaluation of the optimal value of *K* was performed using STRUCTURE HARVESTER, version 6.94 [60], and the output was visualized in DISTRUCT, version 1.0 [61]. Principal coordinates analysis (PCoA), based on the covariance standardized method of pairwise Nei’s genetic distance implemented in GenAIEx, was used to further determine the genetic structure of the studied walnut populations.

## Figures and Tables

**Table 1:** Population genetics summary statistics and PCR amplification rates of 95 *Juglans* individuals from 7 populations at 33 loci.

Locus	*J. sigillata*									*J. regia*						*J. cathayensis*			*J. mandshurica*			Total (*n* = 95)	PCR Amplification Rate
	GD (*n* = 20)			LJPT (*n* = 20)			LBT (*n* = 20)			GLR (*n* = 20)			XYR (*n* = 9)			MGY (*n* = 3)			DTL (*n* = 3)				
	*H*_O_	*H*_E_	*N*_A_	*H*_O_	*H*_E_	*N*_A_	*H*_O_	*H*_E_	*N*_A_	*H*_O_	*H*_E_	*N*_A_	*H*_O_	*H*_E_	*N*_A_	*H*_O_	*H*_E_	*N*_A_	*H*_O_	*H*_E_	*N*_A_	*N*_A_	
JR01	0.000	0.095	2 *	0.200	0.180	2	0.050	0.049	2	0.450	0.546	4	0.000	0.000	1	0.333	0.500	3	0.000	0.000	1	7	100%
JR02	0.588	0.689	5	0.700	0.586	5	0.500	0.685	4 *	0.050	0.386	3 *	0.444	0.568	3	0.667	0.667	4	0.667	0.667	4	9	97%
JR03	0.706	0.685	5	0.300	0.429	3	0.550	0.516	4	0.400	0.584	4 *	0.444	0.494	3	0.333	0.500	3	0.000	0.000	1	7	97%
JR04	0.700	0.754	6	0.550	0.544	4	0.500	0.488	7	0.500	0.738	4 *	0.444	0.642	4	0.000	0.667	3	0.000	0.444	2	12	100%
JR05	0.600	0.530	5	0.650	0.580	5	0.550	0.584	4	0.250	0.219	2	0.111	0.204	3	0.333	0.500	3	0.000	0.444	2	9	100%
JR06	0.400	0.656	4 *	0.400	0.566	4	0.700	0.559	3	0.647	0.554	3	0.556	0.611	3	0.000	0.444	2	0.333	0.278	2	6	97%
JR07	0.450	0.566	4	0.400	0.569	4 *	0.579	0.661	6	0.125	0.443	4 *	0.500	0.602	3	0.000	0.444	2	0.000	0.000	1	7	94%
JR08	0.500	0.526	3	0.400	0.446	4	0.450	0.581	4 *	0.100	0.395	3 *	0.556	0.512	3	1.000	0.500	2	0.000	0.000	1	5	100%
JR09	0.200	0.228	4	0.400	0.448	5	0.313	0.361	3 *	0.125	0.576	4 *	0.556	0.611	3	0.333	0.722	4	0.000	0.444	2	8	92%
JR10	0.200	0.440	5	0.000	0.455	2 *	0.150	0.410	4 *	0.000	0.480	2 *	0.778	0.549	3	0.500	0.625	3	0.333	0.611	3	5	99%
JR11	0.650	0.576	3	0.550	0.696	4	0.650	0.708	5 *	0.400	0.485	3 *	0.667	0.623	4	0.333	0.611	3	0.333	0.278	2	5	100%
JR12	0.100	0.095	2	0.550	0.504	4	0.300	0.329	3	0.650	0.489	2	0.111	0.401	2	0.667	0.667	4	0.667	0.667	3	6	100%
JS02	0.650	0.545	4	0.632	0.536	3	0.500	0.480	2	0.000	0.000	1	0.000	0.000	1	0.000	0.000	1	0.000	0.444	2	7	99%
JS03	0.650	0.731	5	0.400	0.445	5	0.750	0.560	5	0.474	0.411	2	0.111	0.105	2	1.000	0.500	2	0.667	0.611	3	6	98%
JS04	0.550	0.696	5	0.950	0.775	6	0.400	0.700	5 *	0.400	0.471	3	0.444	0.660	3	0.333	0.500	3	0.000	0.000	1	7	100%
JS05	0.650	0.565	3	0.750	0.518	5	0.550	0.526	3	0.000	0.000	1	0.333	0.500	2	0.000	0.000	1	0.000	0.000	1	8	96%
JS06	0.700	0.599	3 *	0.600	0.595	3	0.550	0.511	3	0.000	0.000	1	0.667	0.494	2	0.000	0.000	1	0.000	0.000	1	4	98%
JS07	0.350	0.499	2	0.700	0.495	2	0.550	0.545	3	0.000	0.320	2 *	0.556	0.475	2	0.000	0.444	2	0.000	0.000	1	6	100%
JS09	0.550	0.443	4	0.400	0.341	4	0.500	0.415	5	0.000	0.000	1	0.444	0.426	3	0.333	0.500	3	0.000	0.000	1	6	100%
JS12	0.450	0.421	3 *	0.700	0.579	3	0.550	0.629	3	0.350	0.469	2	0.667	0.648	4	0.000	0.444	2	0.000	0.444	2	8	100%
JS13	0.600	0.585	3	0.500	0.619	5	0.500	0.686	6	0.000	0.000	1	0.667	0.648	3	0.333	0.611	3	1.000	0.611	3	6	99%
JS14	0.650	0.546	4	0.800	0.636	4	0.550	0.659	3	0.000	0.000	1	0.375	0.430	2	0.000	0.000	1	1.000	0.500	2	5	98%
JS15	0.100	0.095	2	0.600	0.670	4	0.600	0.574	5	0.300	0.255	2	0.556	0.512	3	0.000	0.444	2	0.000	0.000	1	6	100%
JS19	0.368	0.499	4 *	0.368	0.716	6 *	0.400	0.713	6 *	0.000	0.000	1	0.000	0.000	1	0.000	0.444	2	0.333	0.278	2	10	97%
JS22	0.650	0.785	6	0.600	0.606	4	0.600	0.718	7	0.000	0.000	1	0.444	0.346	2	0.000	0.444	2	0.333	0.278	2	10	100%
JS28	0.050	0.049	2	0.316	0.359	4	0.200	0.261	3	0.400	0.420	2	0.111	0.105	2	0.333	0.500	3	0.000	0.000	1	5	99%
BFU-Jr277	0.600	0.486	3	0.421	0.486	4	0.400	0.429	3	0.500	0.455	2	0.222	0.346	2	0.000	0.000	1	0.000	0.000	1	5	96%
BFU-Jr38	0.400	0.515	3 *	0.700	0.696	5	0.600	0.581	7	0.316	0.432	2	0.667	0.599	4	0.000	0.000	1	0.000	0.000	1	12	98%
CUJRD102	0.200	0.255	2	0.333	0.292	3	0.316	0.332	2	0.421	0.499	2	0.222	0.198	2	0.333	0.500	3	0.333	0.611	3	6	96%
CUJRD462	0.100	0.185	3	0.550	0.661	5	0.300	0.404	4	0.650	0.489	2	0.222	0.346	2	0.333	0.611	3	0.000	0.444	2	6	100%
JM5446	0.350	0.374	3	0.250	0.301	3	0.350	0.289	2	0.000	0.000	1	0.111	0.105	2	0.333	0.611	3	0.333	0.611	3	8	100%
SSR18	0.850	0.674	8	0.700	0.596	3	0.650	0.569	4	0.400	0.434	3	0.778	0.660	3	0.667	0.500	3	0.333	0.278	2	9	100%
ZMZ7	0.100	0.095	2	0.350	0.503	4	0.750	0.630	6	0.450	0.399	2	0.222	0.346	2	0.333	0.278	2	0.333	0.500	3	8	100%

*N*_A_ = Number of alleles per locus; *H*_O_ = Observed heterozygosity; *H*_E_ = Expected heterozygosity; * = Significant level for deviations from Hardy–Weinberg equilibrium (significance is *p* < 0.05).

**Table 2:** Primer basic information and fluorescence labeling of each multiplex PCR reaction. M1 to M5 are the reaction numbers.

	Primer Pair Sequence (5′–3′)	Repeat Motif	Size Range (bp)	*T*a (°C)	Fluorescence
**M1**				**57**	
JS09	F: TTCGACCGCGTTTCCAGTTA	(TTC)_7_	116–131	56	FAM
	R: CCAGACTCACGGTCAGTTCC				
JS12	F: TCAACATTGGCGAGGTGACA	(TTA)_7_	128–155	55	TAMRA
	R: AGGCAAGTCTACTTCTTTCCCT				
ZMZ7	F: GAACAAATAGACCAGGCACG	(TCC)_7_	215–236	56	TAMRA
	R: TAACGACAACCGATGAAACC				
JR03	F: ATACGGATCTGATGGCATGG	(GAC)_6_	235–268	57	HEX
	R: AGACAGCAATATCCACCCTT				
**M2**				**58**	
CUJRD462	F: TGCTCATTTTCATCCACTATC	(GAA)_8_	250–268	55	HEX
	R: ACTTCCTCTCCTTCCTCTTTC				
JS03	F: TGACGAGGTTTACCAGATGGG	(GAA)_5_	90–105	58	TAMRA
	R: CGTTCTTCTTTCAGAGTGCTGTT				
JS04	F: CATACATATGTGGGTGGCCT	(GAA)_6_	349–367	57	TAMRA
	R: TCCTCCTCTCTCTTCCCTTT				
JS07	F: ACCAGCAGTTCCATGTACGG	(GAG)_9_	111–132	57	HEX
	R: GCTCATGCCATTATCTGCTTCG				
JS19	F: AGATGATTTATGGCAGCCAATGA	(AAG)_7_	216–252	56	FAM
	R: TGCTGGGTAAACGCATGAGT				
JR05	F: GTCGCAAGCTCAGCAAATAA	(AAAG)_8_	194–214	57	HEX
	R: TGTATGTATGGGAGGGGGAT				
JR07	F: TCTTAAGAAGAGCCAATCGC	(ACCA)_5_	303–330	56	FAM
	R: GCTGTGTACCTCTTAGGGTT				
JR11	F: AGCTAGCTCTCAAACAACAAGC	(GCAGTA)_8_	140–164	53	FAM
	R: ACAAACATGGCAACCTTCGTG				
**M3**				**56**	
BFU-Jr38	F: AGCTCCTCAAGCAAGGCTTA	(GAT)_13_	127–145	60	FAM
	R: GTGCATGGAACCACACTCAG				
BFU-Jr277	F: TATTCACCCGGAGGTTTCAG	(GAT)_10_	235–250	61	FAM
	R: CCGAAGCCAGTCGAGTTATC				
JM5446	F: ATGCATGCAGCTCCTACCTC	(CTAG)_5_	221–249	56	HEX
	R: GGACGTGTCCTGGGTTTTCA				
JS05	F: CGGCATTACAGTCGGCAGTA	(GAA)_10_	93–120	57	TAMRA
	R: ACAATTCCCGTGCTGCATCT				
JS15	F: ATCTCCGTGACTCCGCTCCT	(TTG)_5_	352–377	60	TAMRA
	R: ACCCGCCACCATCTTCATCTACCAA				
JR02	F: GTTGGGCTGCCAGAGATTCT	(TTC)_7_	141–168	56	HEX
	R: ACGCTTCATTGGTAAACGAACG				
JR06	F: TTGGAGCCCAATCAAGGATT	(ACAG)_5_	299–319	57	HEX
	R: CACACAGAAAAGACCAGCAG				
JR08	F: ACTCCTGTCACTTGTATGCC	(CACG)_5_	329–359	57	FAM
	R: CCCGAGACATCAGAACCTTT				
JR12	F: GCCTCTCCTCGTGCTCATTT	(GAA)_18_	212–230	56	TAMRA
	R: ACTCGCTACTTTTCAGGCCC				
**M4**				**58**	
CUJRD102	F: GACAGCAGCCTTATTTTGTAAC	(GAG)_8_	169–184	53	HEX
	R: TTCGTCCTCTTCTTCTTCAAC				
JS06	F: CCCTGCATGCAATCAATCACA	(AGT)_5_	96–111	55	TAMRA
	R: ATGGGACGAGTGATGGACTC				
JS14	F: CACATCGAGTGTTTCAAGTGACA	(TGC)_6_	134–149	57	FAM
	R: TGCACATGAGGAATTAACTGCTT				
JS22	F: AAAGTTGCTCCTCAGCTTGG	(ATC)_7_	266–293	56	FAM
	R: TAATTAGCAATGAACAGATGGTGG				
JR04	F: TGTTCTACCATTGCTCCGAA	(TCA)_6_	348–381	57	FAM
	R: ACACCTAGTTAGGAGCTGGA				
JR10	F: TGGGAAGGGATTTCGTGTTGT	(TCTGA)_5_	195–215	56	TAMRA
	R: TAAGGACGCCCATTGCCATT				
**M5**				**55**	
JS02	F: CAACTCTGTGATTGCATGGG	(AAAG)_8_	383–411	57	HEX
	R: GGTAACTCTCATCGCTAGGG				
JS13	F: TCTTGTCAGCATACTAAGCTTGTT	(TTCT)_5_	129–158	56	HEX
	R: ACTAACTGCATATAGGATCAACCA				
JS28	F: AAAGGGTGAAGGAAGAAATTAGGAT	(AAGAG)_5_	316–332	57	HEX
	R: CCAAATTAAGCCAAACATGGTTGC				
JR01	F: GAGCAGCTATGAAGAGGATGA	(AGA)_6_	236–263	57	HEX
	R: CTGAAATTTGTGGGGGTTCC				
JR09	F: ATCACCTGATGTGGAAGCAA	(GAGGA)_5_	359–394	57	TAMRA
	R: CCATAGGACCCATAACGTGA				
SSR18	F: GGAAAGGGATTTGAGGAGAGAT	(TTC)_8_	297–303	60	TAMRA
	R: GAAGAGGAGGAAGAAGAGGAGG				

Note: The bold number indicates the optimal temperature of the multiplex reaction.

## Data Availability

Genomic data for the two *Juglans regia* specimens were downloaded from the National Center for Biotechnology Information (NCBI) database (PRJNA291087 [25] and PRJNA356989 [26]). The raw data for population genetic analysis are presented in Tables S7 and S8.

## Supplementary Materials

The following supporting information can be downloaded at: https://www.mdpi.com/article/10.3390/plants12244101/s1, Figure S1: Ladders of each primer; Table S1: Information about the 1055 primers; Table S2: Locality and voucher for populations of *J. regia*, *J. sigillata, J. cathayensis,* and *J. mandshurica* used in this study; Table S3: Characteristics of the 118 microsatellite loci for *Juglans sigillata*; Table S4: Information about the final 33 primers; Table S5: Information about the 11 redesigned primers; Table S6: Population genetic diversity statistic of 11 populations of four *Juglans* species; Table S7: Raw data for population genetics analysis of 95 individuals; Table S8: Raw data for population genetics analysis of 209 individuals.
